# Pioglitazone Decreases Hepatitis C Viral Load in Overweight, Treatment Naïve, Genotype 4 Infected-Patients: A Pilot Study

**DOI:** 10.1371/journal.pone.0031516

**Published:** 2012-03-07

**Authors:** Mario Chojkier, Hisham Elkhayat, Dina Sabry, Michael Donohue, Martina Buck

**Affiliations:** 1 Department of Medicine, San Diego VA Healthcare System, San Diego, California, United States of America; 2 Department of Medicine, University of California San Diego, La Jolla, California, United States; 3 Biomedical Sciences Program, University of California San Diego, La Jolla, California, United States of America; 4 Clinical Translational Research Institute, University of California San Diego, La Jolla, California, United States of America; 5 Hepatology Department, Theodor Bilharz Research Institute, Giza, Egypt; 6 Hepatology Clinic, Agouza Hospital, Ministry of Health, Giza, Egypt; 7 Department of Medical Biochemistry, Faculty of Medicine, Cairo University, Cairo, Egypt; 8 Division of Biostatistics and Bioinformatics, Department of Family and Preventive, University of California San Diego, San Diego, California, United States of America; Saint Louis University, United States of America

## Abstract

**Background:**

Insulin resistance (IR) is induced by chronic hepatitis C virus (HCV) genotypes 1 and 4 infections. It is not known whether drugs that affect IR such as Pioglitazone and Prednisone also affect serum HCV RNA titers independently of PEG-Interferon-α2/ribavirin treatment. The primary aim was to assess whether Pioglitazone by improving IR and/or inflammation decreases HCV viral load independently of standard of care HCV treatment. A secondary aim was to assess whether Prednisone, a drug that induces insulin resistance and stimulates HCV viral entry and replication in replicon culture systems, increases HCV viral load in this population.

**Methodology/Principal Findings:**

We designed a two-arm, parallel Pilot Study of overweight, treatment naïve genotype 4 HCV-infected patients at a public referral Liver Clinic in Giza, Egypt. The subjects received Pioglitazone (30 mg/day for 14 days) or Prednisone (40 mg/day for 4 days) in a randomized fashion, but the two arms can be considered independent pilot studies. Only changes from baseline within each arm were assessed and no contrasts of the interventions were made, as this was not an aim of the study. Among 105 consecutive HCV genotype 4 patients, 39 were enrolled based on the optimal sample size and power analysis according to the CONSORT statement; 20 to the Pioglitazone group and 19 to the Prednisone group. Pioglitazone was effective in decreasing serum HCV RNA at day-14 (n = 10; difference of means = 205,618 IU/ml; 95% CI 26,600 to 384,600; *P*<0.001). Although Prednisone did increase serum HCV RNA at day-4 (n = 10; change from baseline = −42,786 IU/ml; 95% CI −85,500 to −15,700; *P* = 0.049), the log_10_ HCV RNA titers were statistically not different from baseline day-0.

**Conclusion/Significance:**

This is the first documentation that Pioglitazone decreases the serum HCV RNA titers independently of PEG-Interferon-α2/ribavirin treatment. The novel findings of our Study provide the foundation for basic and clinical investigations on the molecular mechanisms responsible for the Pioglitazone-induced decrease in HCV genotype 4 RNA titers.

**Trial Registration:**

ClinicalTrials.gov NCT01157975

## Introduction

Chronic hepatitis C viral (HCV) infection markedly increased the risk for type 2 diabetes in cohorts with recognized diabetes risk factors [1]. A meta-analysis also found that HCV infection is associated with excess type 2 diabetes risk [2]. Moreover, IR is a specific feature of chronic HCV genotypes 1 and 4 infections since its prevalence is ∼7-fold higher among these patients compared to chronic Hepatitis B-infected patients [3], and changes in IR occur among patients with chronic HCV infection during and after treatment of HCV with PEG-interferon-α and ribavirin [4].

In patients with genotypes 1, 2 and 3 increased IR was associated with decreased sustained virological response (SVR) [5]. Among patients infected with genotype 1, decreases in Homeostasis Model Assessment (HOMA)-IR correlated with obtaining an SVR and increases in HOMA-IR were observed in patients who relapsed [6]. Similarly, IR may correlate with the virological response to standard of care treatment of HCV genotype 4 patients in Egypt [7].

Because Pioglitazone, a peroxisome proliferator-activated receptor (PPAR)-γ agonist, improves insulin sensitivity [8] and it is also effective in controlling liver inflammation in subjects with non-alcoholic steatohepatitis (NASH) [9], it has been used as triple therapy in addition to PEG-interferon-α and ribavirin to treat HCV-infected patients. The study in HCV genotype 1 non-responders was terminated when none of the five patients responded to the triple therapy at week 12 [10]. In contrast, Pioglitazone improved virological response to PEG-interferon α-2b/ribavirin combination therapy in hepatitis C genotype 4 treatment naïve patients with insulin resistance [11].

However, the mechanisms by which Pioglitazone influenced SVR in treatment naïve patients with HCV genotype 4 infection remain unknown. It is unknown whether Pioglitazone modifies the bioavailability of PEG-interferon α-2b/ribavirin or directly or indirectly affects HCV infection and replication.

Therefore, we designed a Pilot Study to assess whether Pioglitazone decreases HCV viral load in overweight HCV genotype 4, treatment naïve patients independently of standard of care PEG-interferon α-2b/ribavirin treatment. A secondary end-point was to analyze whether Prednisone, a drug that induces insulin resistance in susceptible patients [12] and stimulates HCV viral entry and replication in replicon culture systems [13], increases HCV viral load in this population.

There were no data available on the effect of Pioglitazone or Prednisone on HCV RNA viral load in patients. Here, we provide the first documentation that Pioglitazone decreases the serum HCV genotype 4 RNA titers independently of treatment with PEG-Interferon-α2/ribavirin. Prednisone did increase serum HCV RNA viral load at day-4 in this cohort.

## Methods

The protocol for this trial and supporting CONSORT checklist are available as supporting information; see Checklist S1 and Protocol S1.

### Participants

Eligible participants were all overweight (BMI>25 kg/m^2^), given their susceptibility for IR, adults age 25 to 65 years, with active HCV genotype 4 chronic infection (>10,000 IU/ml viral load) who had a liver biopsy within two years of recruitment (added to the protocol), were treatment naïve (never treated for HCV infection) and met the eligibility criteria for the Study. Exclusion criteria included cirrhosis, the presence of other liver diseases, diabetes, pregnancy, significant clinical co-morbidities, and the inability to provide informed consent. Participants were recruited from July 2010 to February 2011 at the primary care referral of subjects of a disadvantaged social-economical group at the Agouza Hospital in Giza, Egypt. The estimated prevalence of Hepatitis C in Egypt is 12% in adults.

### Ethics

Written informed consent was obtained by the medical staff before entry into the Study. The study was registered at the clinicaltrials.gov website (NCT-01157975) and received Human Subjects' Research approval at the University of California, San Diego (UCSD Human Research protections Program) and at the Agouza Hospital, Giza, Egypt (Director's Research Board) before starting the study. The study protocol conforms to the ethical guidelines of the 1975 Declaration of Helsinki.

### Objectives

Because the objective of the Study was to determine whether Pioglitazone and Prednisone treatment would affect the HCV RNA viral load (decrease and increase it, respectively), and these were not a treatment of the HCV infection in this situation, we decided not to use a placebo since we utilized de-identified samples to blinding the investigators analyzing the outcomes.

### Interventions

Eligible subjects were randomly assigned to receive orally Pioglitazone 30 mg/day for 14 days or Prednisone 40 mg/day for 4 days. These medications were taken in the morning. Visits were at screening (up to 30 days before randomization); randomization (day -2); baseline visit (day 0); and follow-up visits for the Pioglitazone group (on days 2, 4, 7 and 14) and the Prednisone group (on days 2 and 4). For safety issues subjects were continued to be seen off protocol at day-7 post-treatment. Blood samples were obtained at each visit for HCV RNA viral load and for inflammatory, adipogenic, and insulin resistance markers. In addition, a 2-hr oral 75 g glucose tolerance test (OGTT) was performed after a 10-hr fast at day-0 before administration of the medication and at the last day of treatment (day-14 for Pioglitazone and day-4 for Prednisone). On all other visits, patients were also fasting. Patients received the first dose of the study drug (oral Pioglitazone 30 mg or Prednisone 40 mg) after blood samples were obtained on day 0. Compliance with the medication was >90% as assessed at each visit.

### Outcomes

The primary outcome with respect to the effect of Pioglitazone in HCV genotype 4 infections was the decrease in HCV RNA viral load at day 14. The secondary outcome with respect to the effect of Prednisone in HCV genotype 4 infections was the increase in HCV RNA viral load at day 4 instead of at day 14 for safety reasons given the potential adverse events associated with longer corticosteroid treatment.

Additional secondary outcome measures were insulin resistance (oral glucose tolerance test; serum insulin, C-peptide; HOMA-IR; insulin sensitivity check index (QUICKI); HOMA-B; sex hormone BG (SHBG); resistin; adiponectin) and liver injury (alanine aminotransferase (ALT); aspartate aminotransferase (AST)) and inflammation (serum interleukin (IL)-8, IL-28B, tumor necrosis factor (TNF), IL-1α, IL-1β, IL-6, IL-8, IL-10, IFN-γ, monocyte chemokine protein (MCP) and hepatocyte growth factor (HGF)).

De-identified blood samples from all the enrolled subjects that received the treatments were analyzed for the standard clinical laboratory tests and for HCV RNA viral load using PCR amplification (COBAS, TACKMAN) (lowest detectable limit: 50 IU/ml).

A multiplex peptide detection system (Quansys Biosciences, Logan, UT) was utilized according to the manufacturer's protocol to determine inflammatory, adipogenic, and insulin resistance markers. Values calculated from individual pixels using the Q-View Imager system. An enzyme-linked immunosorbent assay kit was used to determine human adiponectin according to the manufacturer's protocol (BioVision, Mountain View, CA).

Surrogate indicators of insulin resistance and β-cell function were analyzed as described previously [14]. The indicators of insulin resistance HOMA1-IR and QUICKI were determined. HOMA1-IR = (FSI×FSG)/22.5 where FSI is fasting serum insulin concentration (µU/l) and FSG is fasting serum glucose (mmol/l). QUICKI = 1/[log (fasting insulin-µU/ml)+log (fasting glucose- mg/dl)]. β-Cell function was assessed by the HOMA –B 20.I_0_/(G_0_ -3.5); I_0_ = fasting insulin in µU/ml and G_0_ = fasting glucose in mmol/l [14].

### Sample size

There were no data available on the effect of Pioglitazone or Prednisone on HCV RNA viral load. However, we considered a reduction of 25% in HCV RNA viral load in 14 days to be relevant as proof of principle of the effects of Pioglitazone on HCV RNA titers. To detect a reduction of 25% in HCV RNA in the Pioglitazone group at day-14 assuming a standard deviation of 20% with a two-sided 5% significance level and a power of 90%, a sample size of 9 patients per group completing the study was necessary, and it was decided to enroll at least 19 subjects per group given an anticipated dropout rate of 50% mainly prior to dosing. Because the cultural environment (unwillingness to tell the research staff that they cannot participate in the study) and the social conditions (such as severe problems with transportation and employer's authorization) were expected to prevent a significant proportion of patients to attend the Clinic for follow-up visits after randomization, a larger number of eligible subjects were randomized to achieve the desirable number of subjects completing the Study. To recruit this number of patients a 12-month inclusion period was anticipated. No interim analysis was performed during the trial. An intention-to-treat analysis was conducted in patients who had received ≥1 dose of study medication (10 patients in each group was optimal based on sample size and power analysis).

### Randomization—Sequence generation

Participants were randomly assigned following simple randomization procedures (computerized random numbers) to one of two treatment groups. Independent pharmacists dispensed either Pioglitazone or Prednisone according to a computer generated randomization list.

### Randomization—Allocation concealment

The decision to accept or reject a participant was made, and informed consent was obtained from the participant, in ignorance of the next assignment in the sequence.

### Randomization—Implementation

Block randomization was by a computer generated random number list prepared by a pharmacist with no clinical involvement in the trial. After the research nurse had obtained the subject's consent, she contacted the pharmacist who was independent of the recruitment process for allocation consignment.

### Blinding

Whereas patients and physicians allocated to the intervention groups were aware of the allocated arm, outcome assessors and data analysts of primary and secondary end-points were kept blinded to the allocation.

### Statistical methods

An intention-to-treat analysis was conducted in patients who had received ≥1 dose of study medication. The primary and secondary endpoints were assessed with a paired-sample t-test. We also explored correlations between IL8 and TNFα using Pearson's correlation coefficient with 95% confidence intervals. The significance level was fixed at α = 5% for all tests. All analyses were carried using R version 2.12.2 (2011 Vienna, Austria; http://www.R-project.org).

## Results

### Participant Flow

**Figure 1:**
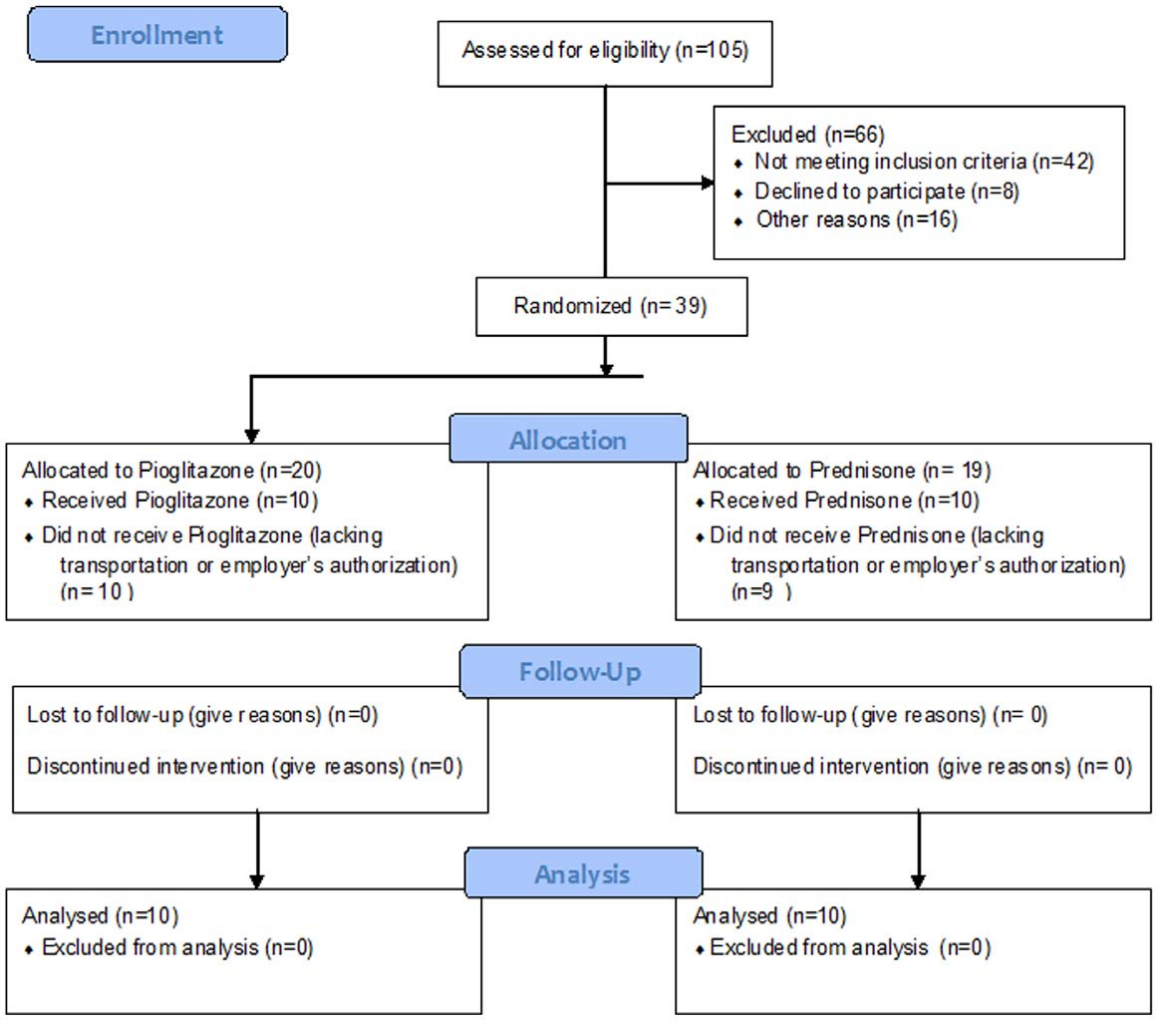
Participant Flow. Patients with chronic Hepatitis C genotype 4 referred to the Hepatology Clinic were assessed for eligibility as described in Methods.

The optimal subjects' recruitment number was pre-determined by sample size and power analysis as described above. One hundredth and five consecutive patients with chronic Hepatitis C genotype 4 referred to the Hepatology Clinic were assessed for eligibility (**Fig. 1**). Among these 105 patients, 66 were excluded because they did not meet the eligibility criteria (42 subjects), they refused to participate (8 subjects), or for other reasons (16 subjects). Therefore, 39 patients were randomized; 20 to the Pioglitazone group and 19 to the Prednisone group. Ten patients in each group received the allocated intervention, while 10 patients in the Pioglitazone group and 9 patients in the Prednisone group were unable to participate because of their inability to attend the Clinic visits. These patients did not receive the allocated intervention (**Fig. 1**). The randomized groups that received the allocated intervention were balanced in regards to age, gender, HCV genotype and titers and the severity of their liver disease (**Table 1**).

**Table 1:** Baseline demographic and clinical characteristics of treated subjects.

	Pioglitazone (N = 10)	Prednisone (N = 10)	*P value*
**Age** (years)	44 (9.3)	42(10.4)	0.592
**Sex** (male)	7 (70%)	8 (80%)	0.429
**Ethnic origin: Arab**	10 (100%)	10 (100%)	1.000
**Height** (cm)	173 (5.8)	175(8.7)	0.458
**Weight** (kg)	88.3 (9.2)	85.7(9.9)	0.550
**BMI** (kg/m^2^)	29.5(1.5)	27.9(1.6)	0.027
**HCV Genotype 4**	10 (100%)	10 (100%)	1.000
**HCV RNA** (log_10_)	5.41(0.80)	5.10(0.92)	0.432
**Albumin** (g/dl)	3.8(0.3)	3.8(0.3)	0.939
**Total bilirubin** (mg/dl)	0.97(0.15)	0.90 (0.16)	0.319
**ALT** (IU/ml)	63 (23)	53 (14)	0.283
**AST**(IU/ml)	54 (17)	52 (11)	0.795
**Metavir score:**			
**A** (inflammation)	A1(n:5) A2(n:5)	A1(n:5) A2(n:5)	1.000
**F**(fibrosis)	F1(n:7); F2(n:3)	F1(n:6); F2(n:4)	0.639

Data are means (SD) or numbers (%).

The patients that did receive the allocated intervention were similar in demographics, ethnicity and degree of liver disease to those that did not receive the allocated intervention (**Table 2**). By chance allocation, the serum ALT was lower in the group that received Prednisone than in the group that did not received the allocated Prednisone (53 vs 69 IU/ml; *P* = 0.012) (**Tables 1** **and** **2**).

**Table 2:** Baseline demographic and clinical characteristics of non-treated subjects.

	Pioglitazone (N = 10)	*P value*	Prednisone (N = 9)	*P value*
**Age** (years)	39 (4.2)	0.139	43(11.2)	0.842
**Sex** (male)	6 (60%)	0.660	5 (55%)	0.277
**Ethnic origin: Arab**	10 (100%)	1.000	9 (100%)	1.000
**Height** (cm)	172 (5.9)	0.706	177(8.1)	0.612
**Weight** (kg)	85.7 (7.6)	0.499	84.8(8.8)	0.837
**BMI** (kg/m^2^)	28.8(2.1)	0.402	28.2(3.7)	0.818
**HCV Genotype 4**	10 (100%)	1.000	9 (100%)	1.000
**ALT** (IU/ml)	68 (10)	0.536	69 (10)	0.012
**AST**(IU/ml)	63 (12)	0.188	57 (8)	0.278
**Metavir score:**				
**A** (inflammation)	A1(n:4); A2(n:6)	0.673	A1(n:4); A2(n:5)	0.821
**F**(fibrosis)	F1(n:5); F2(n:5)	0.388	F1(n:5); F2(n:4)	0.855

Data are means (SD) or numbers (%). *P* values are for comparisons between treated and untreated subjects within the same arm.

No participant was excluded after randomization because they were found not to meet eligibility criteria. All the patients allocated to the interventions (10 in each arm) completed the study (**Fig. 1**).

### Baseline demographic and clinical characteristics

An intention-to-treat analysis was conducted in patients who had received ≥1 dose of study medication; all of these patients had a subsequent efficacy observation (10 patients in each group). As outlined in **Table 1**, all patients were middle age Egyptian Arabs, predominantly males that had HCV genotype 4 with mild or moderate inflammation (A1 and A2, respectively) and mild or moderate fibrosis (F1 and F2, respectively) on their liver biopsy. They had normal albumin and total bilirubin and mild increase in ALT/AST. All subjects were overweight and five were obese (body mass index, BMI; the weight in kg divided by the square of the height in meters was >29 kg/m^2^: 3 in the Pioglitazone group and 2 in the Prednisone group). By chance allocation, the BMI was higher in the group receiving Pioglitazone than in the group receiving Prednisone (29.5 vs 27.9 kg/m^2^; *P* = 0.027) (**Table 1**).

There was no indication that significant chance imbalances had occurred between the two groups. Further, no direct analysis between the two groups was intended for the primary or secondary end points. Rather, a comparison between baseline and post-treatment results within each group was the Pilot Study's intent.

### Effect of Pioglitazone on Serum HCV RNA Titers

Pioglitazone decreased serum HCV RNA titers in overweight HCV genotype 4, treatment naïve patients. In the Pioglitazone-treated group, the log_10_ transformed HCV RNA titers were statistically different from baseline day-0 (5.41+/−0.80) on day-14 (5.24+/−0.79; *P*<0.001) and day-7 (5.30+/−0.78; *P* = 0.009) (**Table 3**), providing evidence that Pioglitazone was effective in decreasing HCV RNA in this cohort of patients. Pioglitazone treatment decreased the HCV RNA from baseline in 9 out of 10 subjects at day-7. The log_10_ HCV RNA values were also decreased by day-4 but they were statistically not different from baseline (5.38+/−0.78; *P* = 0.059). The log_10_ HCV RNA values were not affected at day-2 by Pioglitazone treatment. Further, the decrease in the log_10_ HCV RNA between day-7 and day-14 was highly significant (mean change = 0.063; 95% CI, 0.045; *P*<0.001) (**Table 3**).

**Table 3:** Effects of Pioglitazone on serum HCV RNA titers.

Day	HCV (IU/ml ×10^−3^)	*P value*	*95% CI*	HCV log_10_	*P value*	*95% CI*
**0**	608.2(696.4)			5.41(0.80)		
**2**	586.9(663.9)	0.178	−11.5, 53.9	5.41(0.78)	0.854	−0.029
**4**	531.5(553.6)	0.151	−33.6, 186.9	5.38(0.78)	0.077	−0.002
**7**	455.3(504.4)	0.044	4.8, 300.7	5.30(0.78)	0.001	−0.156
**14**	402.5(452.4)	0.029	26.6, 384.6	5.24(0.79)	<0.001	−0.215

Values are mean (SD) of HCV RNA titers determined as described in Methods.

In spite of the variable baseline HCV RNA viral load, Pioglitazone also decreased significantly the absolute HCV RNA levels at day-14 (mean change = 205,618 IU/ml; *P* = 0.028) and at day-7 (mean change = 152,772 IU/ml; *P* = 0.044) (**Table 3**).

### Effect of Pioglitazone on Indices of Insulin Resistance

Pioglitazone treatment decreased by day-14 fasting serum glucose (Glucose-0) (mean change = 8.2 mg/dL; *P* = 0.024) as well as the 2-hr glucose (Glucose-120 min) after an OGTT (mean change = 18.8 mg/dL; *P* = 0.001) (**Table 4**).

**Table 4:** Effects of Pioglitazone on glucose homeostasis, insulin resistance and secretion.

Test	Day-0	Day-14	*P value*	*95% CI*
**Glucose-0** (mg/dL)	105 (11.4)	96.8 (6.8)	0.024	1.3, 15.1
**Glucose-120** (mg/dL)	120.6 (11.9)	101.8 (7.4)	0.0004	10.9, 26.7
**HOMA-IR**	2.71 (1.61)	2.38 (1.60)	0.326	−0.95
**QUICK-I**	0.339 (0.031)	0.348 (0.036)	0.587	**……**
**HOMA-B**	92.65 (57.5)	115.26 (90.85)	0.52	−99.6, 54.4
**Insulin** (µIU/ml)	10.4 (6.2)	10.1 (7.0)	0.914	−6.2, 6.8
**C-peptide** (nmol/L)	6.4 (9.4)	9.6 (8.5)	0.278	**−**9.5, 3.1
**SHBG** (µg/ml)	3.8 (2.4)	1.7 (2.1)	0.062	−140
**Adiponectin** (µg/ml)	3.19 (1.32)	3.64 (1.71)	0.512	**……‥**
**Resistin** (ng/ml)	2,0 (0.2)	1,7 (0.6)	0.116	−2.0

Values are mean (+/− SD). Tests were performed as described in Methods.

However, Pioglitazone treatment did not affect by day-14 the following surrogate indicators of insulin resistance and secretion: HOMA-IR; QUICK-I; and HOMA-B. In addition, other surrogate indicators of insulin resistance were not affected by Pioglitazone treatment on day-14, including fasting serum insulin; C-peptide; SHBG; resistin; and adiponectin (**Table 4**).

### Effect of Pioglitazone on Indicators of Liver Injury and Inflammation

Elevated serum ALT and AST values are surrogate indicators of liver injury used in patient care and clinical research [9]. Pioglitazone treatment decreased at day-14 serum ALT levels (mean change = 7.5 IU/ml; 95% CI: 2.1 to 12.9; *P* = 0.012) and to a smaller degree serum AST levels (mean change = 2.6 IU/ml; 95% CI: −0.6 to 5.8; *P* = 0.096) (**Table 5**).

**Table 5:** Effects of Pioglitazone on liver injury indicators.

Day	ALT (IU/L)	*P value*	*95% CI*	AST (IU/L)	*P value*	*95% CI*
**0**	62.7 (23.1)			53.6 (15.6)		
**14**	55.2(18.7)	0.0118	2.1, 12.9	51.0 (14.0)	0.0963	−0.6, 5.8

Values are mean (+/− SD). ALT and AST were determined as described in Methods.

It has been suggested that serum IL-8 values are elevated in HCV infection and that these values may reflect the effects of the HCV on *il-8* gene transcription [15]. Pioglitazone increased serum IL-8 levels at day-14 when compared to baseline day-0 values (mean change = −166.8 IU/ml; 95% CI: −300.1 to −33.5; *P* = 0.019) (**Table 6**). Given the possible induction of IL-8 expression by TNF-α [15], we analyzed post-hoc their correlation by a Pearson's correlation coefficient. We found that there was a relatively strong correlation between serum IL-8 and serum TNF-α levels for day-0 and day-14 (n = 20; r = 0.64; 95% CI = 0.28 to 0.85; *P* = 0.002).

**Table 6:** Effects of Pioglitazine on serum cytokines.

Serum Cytokine	Day-0	Day-14	*P value*	*95% CI*
**IL-8** (pg/ml)	181.0 (182.8)	347.8 (123.9)	0.020	−300, −33.5
**IL-28B** (pg/ml)	837.2 (1,068)	1,597 (955.5)	0.060	−1,564, 47
**TNF-α** (pg/ml)	92.9 (139.6)	275.5 (268.5)	0.056	−371.7, 6.6
**IL-1α** (pg/ml)	136.6 (324.4)	223.6 (452.1)	0.415	−317, 143
**IL-1β** (pg/ml)	627.4 (1,046)	724.0 (1,098)	0.748	−760, 566
**IL-2** (pg/ml)	13.9 (11.1)	16.1 (10.1)	0.632	−12.3, 7.9
**IL-4** (pg/ml)	10.9 (4.9)	10.4 (4.3)	0.817	−4.8, 5.9
**IL-6** (pg/ml)	337.9 (454.8)	560.5 (482.1)	0.280	−661, 216
**IL-10** (pg/ml)	32.4 (58.2)	27.6 (44.6)	0.831	−44.7, 54.3
**IFN-γ** (pg/ml)	5.7(4.2)	7.4 (4.6)	0.403	−5.9, 2.6
**MCP-1** (pg/ml)	338.4(151.4)	330.6(157.9)	0.915	155, 170
**HGF** (pg/ml)	1008 (736)	975 (603)	0.914	−665, 732

Values are mean (+/− SD). Serum cytokines were determined as described in Methods.

We found no statistically significant differences between day-0 and day-14 in serum IL-28B, TNF-α, IL-1α, IL-1β, IL-6, IL-8, IL-10, IFN-γ, MCP-1 and HGF (**Table 6**).

### Effect of Prednisone on Serum HCV RNA

Prednisone increased the absolute HCV RNA level at day-2 (mean change = −35,636 IU/ml; 95% CI −64,665 to −6,607; *P* = 0.021) and at day-4 (mean change = −42,786 IU/ml; 95% CI −85,556 to −15.7; *P* = 0.049) (**Table 7**), providing some evidence that Prednisone may be effective in increasing HCV RNA in this cohort of patients for that short period of time. When compared to their baseline day-0 values, eight of 10 subjects had an increase in HCV RNA at day-4. Prednisone treatment also increased HCV RNA from baseline day-0 in 9 of 10 subjects at day-2 (**Table 7**). However, in the Prednisone-treated group, the HCV RNA titers expressed as log_10_ values on day-2 and day-4 were not statistically different from baseline day-0 (**Table 7**).

**Table 7:** Effects of Prednisone on serum HCV RNA titers.

Day	HCV (IU/ml ×10^−3^)	*P value*	*95% CI*	HCV log_10_	*P value*	*95% CI*
**0**	498.0(805.3)			5.10(0.92)		
**2**	533.7(834.4)	0.0215	−64.6,−6.6	5.14(0.94)	0.059	0.08
**4**	540.8(855.2)	0.0499	−85.5,−15.7	5.13(0.92)	0.18	0.03

Values are mean (SD). HCV RNA titers were determined as described in Methods.

### Effect of Prednisone on Indices of Insulin Resistance

Prednisone treatment increased by day-4, when compared to baseline day-0 values, fasting serum glucose (Glucose-0) (mean change = 9.0 mg/dL; 95% CI: 20.4 to −2.4; *P* = 0.108) as well as the 2-hr (Glucose-120 min) after an OGTT (mean change = 11.0 mg/dL; 95% CI: 17.9 to 4.1; *P* = 0.006). Collectively, the CI and t-tests suggest that Prednisone increased serum glucose levels at day-4 (**Table 8**). However, Prednisone treatment did not affect by day-4 other surrogate indicators of insulin resistance and secretion (**Table 8**).

**Table 8:** Effects of Prednisone on glucose homeostasis, insulin resistance and secretion.

Test	Day-0	Day-4	*P value*	*95% CI*
**Glucose-0** (mg/dL)	88.0 (14.7)	97.0 (12.9)	0.107	−20.4, 2.4
**Glucose-120** (mg/dL)	96.4 (10.5)	107.4 (12.3)	0.0055	−17.9, −4.1
**HOMA-IR**	1.73 (1.06)	2.55(1.96)	0.162	−1.06
**QUICK-I**	0.358(0.022)	0.345 (0.032)	0.275	
**HOMA-B**	95.48 (49.35)	115.86 (95.96)	0.623	−20.38
**Insulin** (µIU/ml)	7.5(3.9)	10.5 (8.2)	0.374	−10.2, 4.4
**C-peptide** (nmol/L)	11.3 (8.9)	4.1 (1.3)	0.061	−0.5, 14.9
**SHBG** (µg/ml)	3.4(2.5)	2.7 (2.2)	0.591	−2.4
**Adiponectin** (µg/ml)	5.9 (8.2)	2.6 (2.1)	0.306	−4.5
**Resistin** (ng/ml)	3.4 (2.5)	2.7 (2.2)	0.591	−2.4

Values are mean (+/− SD). Tests were performed as described in Methods.

### Effect of Prednisone on Indicators of Liver Injury and Inflammation

Prednisone treatment did not affect at day-4 either serum ALT, AST, IL-8 or other inflammatory mediators (**Tables 9** **and** **10**).

**Table 9:** Effects of Prednisone on liver injury indicators.

Day	ALT (IU/L)	*P value*	*95% CI*	AST (IU/L)	*P value*	*95% CI*
**0**	53.2 (14.4)			52.0(11.3)		
**4**	55.7 (15.7)	0.080	5.4, −0.4	52.2 (12.8)	0.896	3.6, −3.2

Values are mean (+/− SD). ALT and AST were determined as described in Methods.

**Table 10:** Effects of Prednisone on serum cytokines.

Serum Cytokine	Day-0	Day-4	*P value*	*95% CI*
**IL-8** (pg/ml)	123.1 (163.5)	159.5 (185.1)	0.353	−123, 50
**IL-28B** (pg/ml)	485.9(876.4)	610.8 (857.7)	0.120	−292, 42
**TNF-α**(pg/ml)	146.6 (268.8)	146.1 (284.4)	0.996	−275, 276
**IL-1α** (pg/ml)	80.1 (208.3)	19.2(38.3)	0.450	−119, 240
**IL-1β** (pg/ml)	92.7 (120.3)	144.8 (293.0)	0.682	−348, 244
**IL-2** (pg/ml)	11.8 (9.8)	11.3 (9.6)	0.742	−3.1, 4.2
**IL-4** (pg/ml)	19.3 (19.1)	10.9 (7.2)	0.329	−10.5, 27.3
**IL-6** (pg/ml)	292.8 (445.7)	359.9 (501.4)	0.492	−282, 149
**IL-10** (pg/ml)	67.4 (106.8)	30.8 (51.2)	0.144	−16.1, 89.4
**IFNγ** (pg/ml)	13.9 (24.9)	4.9 (4.3)	0.342	−11.9, 29.9
**MCP-1** (pg/ml)	232.7 (126.1)	266.8 (112.8)	0.469	−139, 71
**HGF** (pg/ml)	1345 (800)	1022 (681)	0.483	−711, 1357

Values are mean (+/− SD). Serum cytokines were determined as described in Methods.

### Adverse Events

There were no serious adverse events in either treatment group. Mild or moderate adverse events unrelated to the allocated treatment occurred in 20% of subjects in the Prednisone group and in 30% of the subjects in the Pioglitazone group. There were no significant changes from baseline during treatment in safety laboratory tests in either treatment group.

## Discussion

It was not known whether Pioglitazone and Prednisone affect serum HCV genotype 4 RNA titers independently of PEG-Interferon-α2/ribavirin treatment. This Study documented for the first time that Pioglitazone (30 mg daily) decreases the serum HCV RNA titers after a 14-day treatment in overweight subjects with treatment naïve, chronic HCV genotype 4 infections. The statistical differences between the mean at day-0 (baseline) and day-14 were highly significant whether the serum HCV RNA was analyzed as IU/ml or log_10_ values (**Table 3**). Pioglitazone treatment also decreased the HCV genotype 4 titers on day-4 and day-7, suggesting a rapid effect on HCV RNA titers. The comparable mean decrease in HCV RNA for the first week and for the second week indicates a continuing effect during the 14-day treatment.

It remains to be studied whether the rate of decrease of serum HCV RNA titers continues after day-14 in the Pioglitazone group. If the effects of Pioglitazone on in HCV genotype 4 RNA titers would continue at the same rate as for the first 2 weeks, it would achieve a clinically significant decrease of ∼1 log_10_ in HCV RNA titers by week-12, an important predictive timeline for a sustained virological response in HCV treatments [16]. Given the relative safety of Pioglitazone administration [9],[17], it could be, as a co-adjuvant, a valuable therapeutic effect for difficult to treat HCV genotype 4-infected patients.

This Study also documented for the first time that Prednisone (40 mg daily) increases the serum HCV RNA titers after a 4-day treatment of HCV genotype 4 overweighed patients (**Table 7**). Our results with Prednisone treatment are congruent with the recently reported increased HCV replication by corticosteroids in the Huh-7 replicon system [13], but whether in HCV genotype 4-infected patients, this reflects a direct corticosteroid effect on HCV replication, an indirect effect mediated by insulin resistance, or another mechanism is unknown. Because the Huh-7 replicon system uses de-differentiated, tumoral liver cells that have higher expression of glucose metabolism enzymes (glucose-6-phosphate 1-dehydrogenase and isocitrate dehydrogenase) and of the mitochondrial dicarboxylate carrier [18], it may not be suitable for the evaluation of interaction between glucose homeostasis and HCV replication. To minimize potential adverse events associated with the administration of Prednisone, its use was limited to 40 mg/day for a 4-day treatment, a relatively safe and frequent standard of care practice for some clinical conditions [19]. It remains to be determined whether a more prolonged corticosteroid treatment in HCV-infected patients has detrimental effects on RNA titers of different HCV genotypes.

The subjects' recruitment number was pre-determined by the sample size and power analysis. An appropriate sample size was utilized in this study, which achieved the primary and secondary end-points with a relatively small cohort of patients. If the sample size of the study were unnecessarily large, then the risks to the subjects would have been excessive according to the CONSORT statement [20]. An intention-to-treat analysis was conducted in patients who had received ≥1 dose of study medication; all of these subjects completed the study and had a subsequent efficacy observation. Because these randomized arms were not treatments of the HCV infection in this Study, and because during chronic infection, the level of serum HCV RNA is in a steady state with only minor fluctuations in untreated patients [21], instead of using demanding placebo arms, we utilized randomization as well as de-identified samples to blinding the investigators analyzing the outcomes. These approaches minimize biases in executing the study and in analyzing the outcomes according to the CONSORT statement [20].

Triple combination therapy of Pioglitazone/PEG-Interferon-α2/ribavirin has been used for previously treated HCV genotype 1 infection unsuccessfully in Switzerland [10] and in the USA [22], [23] or for treatment naive HCV genotype 4 infection successfully in Egypt [11]. It is unknown whether the beneficial effects of Pioglitazone together with PEG-Interferon-α2/ribavirin are circumscribed to HCV genotype 4 or to treatment naïve HCV of various genotypes. Although, the relatively low 15 mg Pioglitazone dose used in the INSPIRED-HCV trial in Switzerland [10] could have been insufficient to achieve efficacy, higher Pioglitazone doses (30 mg or 45 mg per day) have been utilized in HCV genotype 1 infection trials together with PEG-Interferon-α2/ribavirin without improving the sustained virological response [22], [23]. These higher Pioglitazone doses (30 mg or 45 mg per day) have been successfully utilized to prevent the development of type 2 diabetes in patients with impaired glucose tolerance in the USA [8], in the treatment of HCV genotype 4 treatment naive patients together with PEG-Interferon-α2/ribavirin in Egypt [11] and in our Study. Further, the known effects of Pioglitazone and related PPARγ agonists on glucose homeostasis [8],[12] and as documented in our Study, and their effects on serum HCV RNA titers (**Table 3**) suggest, but does not prove, that improving insulin sensitivity lowers HCV RNA titers. This has been proposed by Harrison on the basis that patients with QUICK-I values <0.35 (equivalent to HOMA-IR>1.8) have significantly higher HCV titers than patients with QUICK-I values >0.35 [24]. Further, treating patients with hepatitis C genotype 1 and insulin resistance using metformin improves insulin sensitivity and increases SVR rate in patients who reached HOMA lower than 2 at week 24 of therapy and in women, in whom the therapy doubled the SVR rate [25].

The findings of our Study support the notion that sequential treatment with Pioglitazone and PEG-Interferon-α2/ribavirin rather than concomitant administration should be considered in the treatment of chronic HCV genotype 4 infections, as suggested by Serfaty and coworkers [26].

The beneficial effects of Pioglitazone treatment on glucose homeostasis have been well documented in patients with impaired glucose tolerance [8], and it was also reported in triple combination therapy of Pioglitazone/PEG-Interferon-α2/ribavirin in patients with HCV genotype 1 or 4 [10],[11]. In our Study, Pioglitazone treatment decreased by day-14 and Prednisone increased by day-4 fasting serum glucose as well as the 2-hr glucose after an OGTT (**Table 4** **and** **8** **)**. However, when compared to baseline day-0 values, neither Pioglitazone treatment at day-14 nor Prednisone treatment at day-4 affected HOMA-IR; QUICK-I; and HOMA-B, surrogate indicators of insulin resistance and secretion (**Tables 4** **and** **8**). It would probably require a more prolonged treatment with Pioglitazone and Prednisone to induce changes in these surrogate indicators of insulin resistance and secretion [26].

Elevated serum ALT and AST values are surrogate indicators of liver injury used in patient care and clinical research [9]. Pioglitazone treatment decreased at day-14 serum ALT levels and to a smaller degree serum AST levels (**Table 5**). Additional insights into the mechanisms of Pioglitazone on liver injury in chronic HCV infection would require assessment of liver biopsies before and after prolonged treatment since the effects on serum ALT were clinically modest at day-14. Prednisone treatment did not affect at day-4 serum ALT or AST levels (**Table 9**).

Many experimental and clinical studies have shown that PPARγ signaling has anti-inflammatory properties in the liver [27]. Interestingly, HCV infection in Huh-7 cells and in humans inhibits PPARγ expression and function [27]. Two studies suggested therapeutic effects of bezafibrate, a non-selective agonist of PPAR α/δ/γ, during chronic HCV infection. Patients with HCV genotype 1 and 2 unresponsive to interferon therapy were treated for 8 weeks or 6 months with bezafibrate [28], [29]. At endpoint, HCV viral load (−0.5 log_10_) and ALT were statistically significantly decreased, suggesting anti-inflammatory and antiviral activities of the PPAR α/δ/γ signaling pathways in patients with chronic HCV infection. Given the non-selective nature of the PPAR α/δ/γ agonist, it is difficult to determine whether induction of PPARγ pathways occurred and played a role in the decreased HCV RNA.

Pioglitazone also has shown improvement in liver injury and inflammation in patients with non-alcoholic steatohepatitis [9] and this has been attributed to the beneficial effects of Pioglitazone on insulin signaling [8]. An alternative explanation is a possible direct effect of Pioglitazone either as a PPARγ agonist or through other mechanisms on liver injury, independently of its insulin sensitizing actions [8]. Indeed, PPARγ agonists induce alternative, anti-inflammatory M2 macrophage activation [30], possibly linking insulin sensitivity with decreased liver injury in patients with HCV infection. At the molecular level, insulin resistance is promoted by a transition from an anti-inflammatory macrophage M2 state maintained by STAT6 and PPARγ to a pro-inflammatory macrophage M1 state driven by C/EBPβ and NF-κB activation and other transcription factors that play important roles in innate immunity [30]. Relevant to the management of HCV infected patients, liver fibrogenesis is expected to be ameliorated by PPARγ signaling [31] as well as by decreased inflammatory pathways arising from M2 macrophages [32] and resulting in decreased activation of hepatic stellate cells [33].

Pioglitazone increased serum IL-8 levels at day-14 when compared to baseline day-0 values (**Table 6**). Serum IL-8 values are elevated in HCV infection [34] and these values may reflect the effects of the HCV on *il-8* gene transcription as well as its induction by TNFα [15]. We found that there was a relatively strong correlation between serum IL-8 and serum TNF-α levels for day-0 and day-14. Although it has been suggested that elevated levels of serum IL-8 may be associated with HCV resistance to Interferon-α2 therapy, the clinical or biological significance of the elevated serum IL-8 in HCV genotype 4 patients treated with Pioglitazone remains to be determined.

Prednisone did not affect serum IL-8 levels at day-4 when compared to baseline day-0 values (**Table 10**). We found no differences between day-0 and day-14 in the Pioglitazone group and day-4 in the Prednisone group in other inflammatory markers such as serum IL-28B, TNF-α, IL-1α, IL-1β, IL-6, IL-8, IL-10, IFN-γ, MCP-1 and HGF (**Tables 6** **and** **10**). The lack of changes in IL-28B (IFN-γ3) is relevant since genetic variation in *IL-28B* affects the spontaneous clearance of HCV in patients [35].

Our study has some limitations: i] there were, as expected, clinically modest decreases in HCV RNA at day-14 in the Pioglitazone group; ii] the relatively small cohort could introduce potential biases; and iii] the findings may not be applicable for patients with HCV genotype 4 in other ethnic groups

It remains to be elucidated whether the effects of Pioglitazone also occur in HCV genotype 4 infections in previously treated patients. The novel findings of our Study provide the foundation for basic and clinical investigations on the molecular mechanisms responsible for the Pioglitazone-induced decrease in HCV genotype 4 RNA titers. Future research should characterize whether the effects of Pioglitazone on HCV genotype 4 RNA titers are mediated indirectly by insulin signaling pathways or directly by affecting HCV genotype 4 hepatocyte entry and/or replication.

## Supporting Information

Checklist S1 **CONSORT Checklist.** CONSORT 2010 checklist of information to include when reporting a randomised trial.
(DOC)

Protocol S1 **Trial Protocol.**
(DOC)
