# Supplementary material for: Pioglitazone Decreases Hepatitis C Viral Load in Overweight, Treatment Naïve, Genotype 4 Infected-Patients: A Pilot Study
Source: PLoS One. 2012 Mar 7;7(3):e31516. doi: 10.1371/journal.pone.0031516 (PMC3296686; doi:10.1371/journal.pone.0031516)
Supplement: Protocol S1 — Trial Protocol. (DOC) [file pone.0031516.s002.doc]

**Study the Relationship Between Obesity and Hepatitis C Replication**

This study is ongoing, but not recruiting participants.

Study NCT01157975 Information provided by University of California, San Diego

First Received on July 6, 2010. Last Updated on November 23, 2011

| **Tracking Information** | | | | |  |
| --- | --- | --- | --- | --- | --- |
| **First Received Date ICMJE** | July 6, 2010 | | | |  |
| **Last Updated Date** | November 23, 2011 | | | |  |
| **Start Date ICMJE** | October 2008 | | | |  |
| **Estimated Primary Completion Date** | February 2012 (final data collection date for primary outcome measure) | | | |  |
| **Current Primary Outcome Measures ICMJE  (submitted: November 23, 2011)** | HCV RNA [ Time Frame: 2 weeks ] [ Designated as safety issue: No ]  Only in the Pioglitazone group | | | |  |
| **Original Primary Outcome Measures ICMJE  (submitted: July 6, 2010)** | HCV viral load [ Time Frame: 2 weeks ] [ Designated as safety issue: Yes ] | | | |  |
| **Change History** | [Complete list of historical versions of study NCT01157975 on ClinicalTrials.gov Archive Site](../../../../ct2/archive/NCT01157975) | | | |  |
| **Current Secondary Outcome Measures ICMJE  (submitted: November 23, 2011)** | HCV RNA [ Time Frame: Day 4 ] [ Designated as safety issue: No ]  Only in the Prednisone group  Serum indicators of insulin resistance (fasting glucose, insulin, lipids and serum retinol binding protein-4); adiponectins and inflammatory cytokines. [ Time Frame: Day 14 (Pioglitazone) and Day 4 (Prednisone) ] [ Designated as safety issue: Yes ]  ALT and AST [ Time Frame: Day 14 (Pioglitazone) and Day 4 (Prednisone) ] [ Designated as safety issue: Yes ] | | | |  |
| **Original Secondary Outcome Measures ICMJE  (submitted: July 6, 2010)** | Serum indicators of insulin resistance (fasting glucose, insulin, lipids and serum retinol binding protein-4); adiponectins and inflammatory cytokines. [ Time Frame: 2 weeks ] [ Designated as safety issue: Yes ] | | | |  |
|  |  | | | |  |
| **Descriptive Information** | | | | |  |
| **Brief Title ICMJE** | Study the Relationship Between Obesity and Hepatitis C Replication | | | |  |
| **Official Title ICMJE** | A Randomized, Partially Blinded, Pilot Study of the Effects of Pioglitazone on HCV RNA in Overweight Subjects With Chronic HCV Genotypes 1 or 4 Infection. | | | |  |
| **Brief Summary** | Patients with chronic hepatitis C viral infection (HCV) and with a BMI greater than 25Kg/m2 are refractory to medical treatment. Also, HCV replication seems to be affected when modeling insulin resistance in replicon cell culture systems.  PPARγ -agonist (Pioglitazone) is effective in controlling liver inflammation in obese subjects with non-alcoholic steatohepatitis (NASH) and also improving insulin sensitivity. Therefore, we hypothesize that improving insulin resistance and /or inflammation may affect HCV replication and viral kinetics. Independently of PPARγ pathways, Prednisone may increase HCV viral kinetics . | | | |  |
| **Detailed Description** | This is a randomized, two arm clinical trial. The investigators performing the primary and secondary endpoints are blinded to subject identifiers and arm identifiers.  Subject's screening for HCV Genotype 4 started in Agouza Hospital in July 2010 and ended in February, 2011. No recruitment has occurred for HCV Genotype 1. | | | |  |
| **Study Type ICMJE** | Interventional | | | |  |
| **Study Phase** | Phase II | | | |  |
| **Study Design ICMJE** | Allocation: Randomized Intervention Model: Parallel Assignment Masking: Open Label | | | |  |
| **Condition ICMJE** | Hepatitis C | | | |  |
| **Intervention ICMJE** | Drug: Pioglitazone  Pioglitazone will be taken at a dose of 30 mg for up to 14 days  Other Name: ACTOS  Drug: Prednisone  Prednisone will be taken at a dose of 40 mg for up to 4 days  Other Name: PREDNISONE | | | |  |
| **Study Arms** | Experimental: Pioglitazone  Intervention: Drug: Pioglitazone  Experimental: Prednisone  Intervention: Drug: Prednisone | | | |  |
| **Publications *** |  | | | |  |
| * Includes publications given by the data provider as well as publications identified by ClinicalTrials.gov Identifier (NCT Number) in Medline. | | | | |  |
|  |  | | | |  |
| **Recruitment Information** | | | | |  |
| **Recruitment Status ICMJE** | Active, not recruiting | | | |  |
| **Estimated Enrollment ICMJE** | 40 | | | |  |
| **Estimated Completion Date** | September 2012 | | | |  |
| **Estimated Primary Completion Date** | February 2012 (final data collection date for primary outcome measure) | | | |  |
| **Eligibility Criteria ICMJE** | Inclusion Criteria:  Infection with HCV genotype 1 or 4 (subjects infected with multiple genotypes are not eligible)  BMI greater than 25 Kg/m2  HCV-infected subjects naïve to treatment: subjects who either have never been treated for HCV infection or who previously received HCV treatment ending more than 3 months prior to enrollment for not longer than 2 weeks  Plasma HCV RNA concentration of >10,000 IU/mL at the screening evaluation  Exclusion Criteria:  Previous intolerance to Pioglitazone, Rosiglitazone, Troglitazone or corticosteroids  Women who are pregnant or breastfeeding  History of diabetes mellitus requiring treatment other than diet  Decompensated liver disease or other known causes of liver disease including, but not limited to autoimmune hepatitis, Wilson's disease, hemochromatosis, primary biliary cirrhosis, schistosomiasis, sclerosing cholangitis, alcohol- or drug-induced liver disease, or alpha-one antitrypsin deficiency  Concurrent hepatitis B virus (HBV) infection  Known immunodeficiency disease, autoimmune disorders or active gastrointestinal disease  Abuse of alcohol or illicit drugs within 6 months before enrollment  Use of an investigational drug within 4 weeks before the screening visit or during the screening period.  Use of systemic immunosuppressants  History of poorly controlled psychiatric disease or poorly controlled pulmonary disease | | | |  |
| **Gender** | Both | | | |  |
| **Ages** | 18 Years to 65 Years | | | |  |
| **Accepts Healthy Volunteers** | No | | | |  |
| **Contacts ICMJE** | Contact information is only displayed when the study is recruiting subjects | | | |  |
| **Location Countries ICMJE** | United States, Egypt | | | |  |
|  |  | | | |  |
| **Administrative Information** | | | | |  |
| **NCT Number ICMJE** | NCT01157975 | | | |  |
| **Other Study ID Numbers ICMJE** | 060913 | | | |  |
| **Has Data Monitoring Committee** | No | | | |  |
| **Responsible Party** | Mario Chojkier, University of California, San Diego | | | |  |
| **Study Sponsor ICMJE** | University of California, San Diego | | | |  |
| **Collaborators ICMJE** |  | | | |  |
| **Investigators ICMJE** | Principal Investigator: | Mario Chojkier, MD | UCSD |  | |
|  | Principal Investigator: | Martina Buck, PhD | UCSD |  | |
|  | Principal Investigator: | Hesham Elkhayat, MD | Cairo University, Egypt |  | |
| **Information Provided By** | University of California, San Diego | | | |  |
| **Verification Date** | November 2011 | | | |  |
